# Reorganization of Corticospinal Projections after Prominent Recovery of Finger Dexterity from Partial Spinal Cord Injury in Macaque Monkeys

**DOI:** 10.1523/ENEURO.0209-23.2023

**Published:** 2023-08-04

**Authors:** Masahiro Sawada, Kimika Yoshino-Saito, Taihei Ninomiya, Takao Oishi, Toshihide Yamashita, Hirotaka Onoe, Masahiko Takada, Yukio Nishimura, Tadashi Isa

**Affiliations:** 1 Department of Developmental Physiology, National Institute for Physiological Sciences, Okazaki 444-8585, Japan; 2 Department of Neurosurgery, Graduate School of Medicine, Kyoto University, Kyoto 606-8501, Japan; 3 Systems Neuroscience, Primate Research Institute, Kyoto University, Inuyama 484-8506, Japan; 4 Core Research for Evolutional Science and Technology, Japan Science and Technology Agency, Saitama 332-0012, Japan; 5 Department of Molecular Neuroscience, Graduate School Medicine, Osaka University, Suita 565-0871, Japan; 6 Human Brain Research Center, Graduate School of Medicine, Kyoto University, Kyoto 606-8507, Japan; 7 Neural Prosthetics Project, Tokyo Metropolitan Institute of Medical Science, Tokyo 156-8506, Japan; 8 The graduate University for Advanced Studies (SOKENDAI), Hayama 240-0193, Japan; 9 Precursory Research for Embryonic Science and Technology, Japan Science and Technology Agency, Saitama 332-0012, Japan; 10 Department of Neuroscience, Graduate School of Medicine, Kyoto University, Kyoto 606-8501, Japan; 11 Institute for the Advanced Study of Human Biology (WPI-ASHBi), Kyoto University, Kyoto 606-8501, Japan

**Keywords:** spinal cord injury, finger dexterity, corticospinal tract, functional recovery, sprouting, primate

## Abstract

We investigated morphologic changes in the corticospinal tract (CST) to understand the mechanism underlying recovery of hand function after lesion of the CST at the C4/C5 border in seven macaque monkeys. All monkeys exhibited prominent recovery of precision grip success ratio within a few months. The trajectories and terminals of CST from the contralesional (*n* = 4) and ipsilesional (*n* = 3) hand area of primary motor cortex (M1) were investigated at 5–29 months after the injury using an anterograde neural tracer, biotinylated dextran amine (BDA). Reorganization of the CST was assessed by counting the number of BDA-labeled axons and bouton-like swellings in the gray and white matters. Rostral to the lesion (at C3), the number of axon collaterals of the descending axons from both contralesional and ipsilesional M1 entering the ipsilesional and contralesional gray matter, respectively, were increased. Caudal to the lesion (at C8), axons originating from the contralesional M1, descending in the preserved gray matter around the lesion, and terminating in ipsilesional Laminae VI/VII and IX were observed. In addition, axons and terminals from the ipsilesional M1 increased in the ipsilesional Lamina IX after recrossing the midline, which were not observed in intact monkeys. Conversely, axons originating from the ipsilesional M1 and directed toward the contralesional Lamina VII decreased. These results suggest that multiple reorganizations of the corticospinal projections to spinal segments both rostral and caudal to the lesion originating from bilateral M1 underlie a prominent recovery in long-term after spinal cord injury.

## Significance Statement

Previous studies have shown that dexterous finger movements recover prominently after lesion of the corticospinal tract (CST) at the mid-cervical segments through rehabilitative training in macaque monkeys. Here, we show reorganization of the CST including sprouting of axons originating from the contralesional and ipsilesional motor cortex in the gray matter both caudal and rostral to the lesion, including a re-direction of the CST to hand motoneurons in the monkeys 5–29 months after the lesion. Thus, multiple mechanisms of reorganization of CST axons underlie the recovery of impaired cortico-motoneuronal pathways for the long-term recovery of finger dexterity.

## Introduction

Hand use is a critical component in our daily life, and recovery of hand function is a major issue for patients with neuronal damage such as spinal cord injury (SCI; Anderson, 2004). Investigating the neuroanatomical basis of recovery in an animal model that shows prominent recovery of finger dexterity is critical for future development of therapeutic strategies to promote the recovery of hand functions. However, the recovery of finger dexterity has been shown to be difficult after large injuries such as hemisection (Galea and Darian-Smith, 1997; Rosenzweig et al., 2010) or subhemisection without drug treatment (Nakagawa et al., 2015). In this regard, it should be noted that macaque monkeys with lesions limited to the dorsolateral funiculus (DLF) which transected the lateral corticospinal tract (l-CST) and rubrospinal tract show prominent recovery of precision grip in several weeks after injury (Sasaki et al., 2004), and our laboratory has been investigating the mechanisms of recovery in this model (Isa, 2017, 2019).

Direct cortico-motoneuronal connections, i.e., the monosynaptic pathway from corticospinal neurons to spinal motoneurons, play a pivotal role in the control of dexterous finger movements in primates (Lawrence and Kuypers, 1968; Kuypers, 1982; Lemon, 2008). Conversely, our previous series of reports documented that indirect cortico-motoneuronal pathways mediated by propriospinal neurons (PNs) located in spinal segments rostral to motoneurons exist in primates, are connected to motoneurons, and partially contribute to the control of dexterous finger movements (Alstermark et al., 1999, 2011; Isa et al., 2006; Kinoshita et al., 2012). Studies on the C4/C5 DLF lesion model demonstrated that the indirect cortico-motoneuronal connections via C3–C4 PNs mediating the command for dexterous finger movements were strengthened and contributed to the prominent recovery of dexterous finger control (Sasaki et al., 2004; Tohyama et al., 2017). Functional reorganization has also been demonstrated at the cortical level. After monkeys showed full recovery of finger dexterity from the C4/C5 DLF lesion, multiple motor-related cortical areas on bilateral sides which are origins of CST showed increased activity (Nishimura et al., 2007; Sawada et al., 2015; Suzuki et al., 2020). These observations suggest that plastic changes in the corticospinal connections from bilateral motor cortices are involved in prominent functional recovery, however it remains unclear whether these functional changes are accompanied by morphologic changes in the corticospinal projections.

Previous studies revealed that the CST exhibits substantial sprouting both caudal and rostral to the lesion after the recovery of hand function in the hemisection/subhemisection models of SCI (Fouad et al., 2001; Freund et al., 2006; Rosenzweig et al., 2010; Nakagawa et al., 2015). However, in these models, the recovery of dexterity was limited because of the large lesion of the spinal cord. Therefore, a quantitative analysis of the reorganization of CST in the partial SCI model with prominent recovery and comparison with the results of hemisection/subhemisection model with poor recovery may be valuable for understanding the key pathway for prominent recovery, which would be the target pathway for neuromodulation therapy. For this purpose, we investigated quantitatively the distribution of CST axons and their terminals in the white matter and each lamina of the gray matter rostral and caudal to the lesion limited to the DLF at the C4/C5 border in macaque monkeys that showed prominent recovery of dexterous finger movements.

## Materials and Methods

### Statement of ethics

All applicable institutional and governmental regulations concerning the ethical use of animals were followed during the course of this research. The experiments were subjected to prior reviews by the ethical committee of the National Institutes of Natural Science and were performed in accordance with the guideline of the Ministry of Education, Culture, Sports, Science, and Technology (MEXT) of Japan and National Institutes of Health *Guide for the Care and Use of Laboratory Animals*.

### Animals

Seven male macaque monkeys (*Macaca fuscata*; Di, To, Al, Gr, De, As, Fu, ranging from 3.6 to 8.6 kg; Table 1) were used in this study. To investigate the anatomic changes induced during functional recovery after SCI, the data obtained from the monkeys with SCI were compared with data from the five nonlesioned control monkeys [Mo-1 (male), Mo-2 (female), Mo-3 (female), Mo-4 (female), Mo-5 (male)] reported in our previous study (Yoshino-Saito et al., 2010).

**Table 1.** Experimental time course and injection sites of BDA in each monkey whose data were used in this manuscript

Name	Injected side	Side to lesion	Days to injection	Survival time after injection (d)	Total survival time (d)	N of tracks	N of injection sites	Injection	Injected amount (μl/site)
Di	Right	Contra	155	75	230	6	15	1.5, 3.0	0.5
								4.5	
To	Right	Contra	563	114	677	8	20	1.5, 3.0	0.5
								4.5	
Al	Right	Contra	278	93	371	7	18	1.5, 3.0	0.5
								4.5	
Gr	Right	Contra	277	178	455	6	12	1.5, 3.0	0.5
								4.5	
De	Left	Ipsi	107	42	149	4	8	3.5, 5.0	0.5
As	Left	Ipsi	124	30	154	4	8	3.5, 5.0	0.5
Fu	Left	Ipsi	731	139	870	7	18	2.0, 5.0	0.5
								8	
Mo1	Right			104		5	15	2.0, 4.0	
								6	
Mo2	Right			103		5	15	2.0, 4.0	
								6	
Mo3	Right			80		4	12	2.0, 4.0	
								6	
Mo4	Right			92		7	21	2.0, 4.0	
								6	
Mo5	Right			94		5	15	2.0, 4.0	
								6	

A 0.5-μl solution of biotinylated dextran amine [BDA; Molecular Probes, 10,000 MW, 10% dissolved in 0.01% phosphate buffer (pH 7.3)] was injected using a 10-μl Hamilton microsyringe. d: day.

### Behavioral training and functional assay of finger dexterity

**Figure 1.**
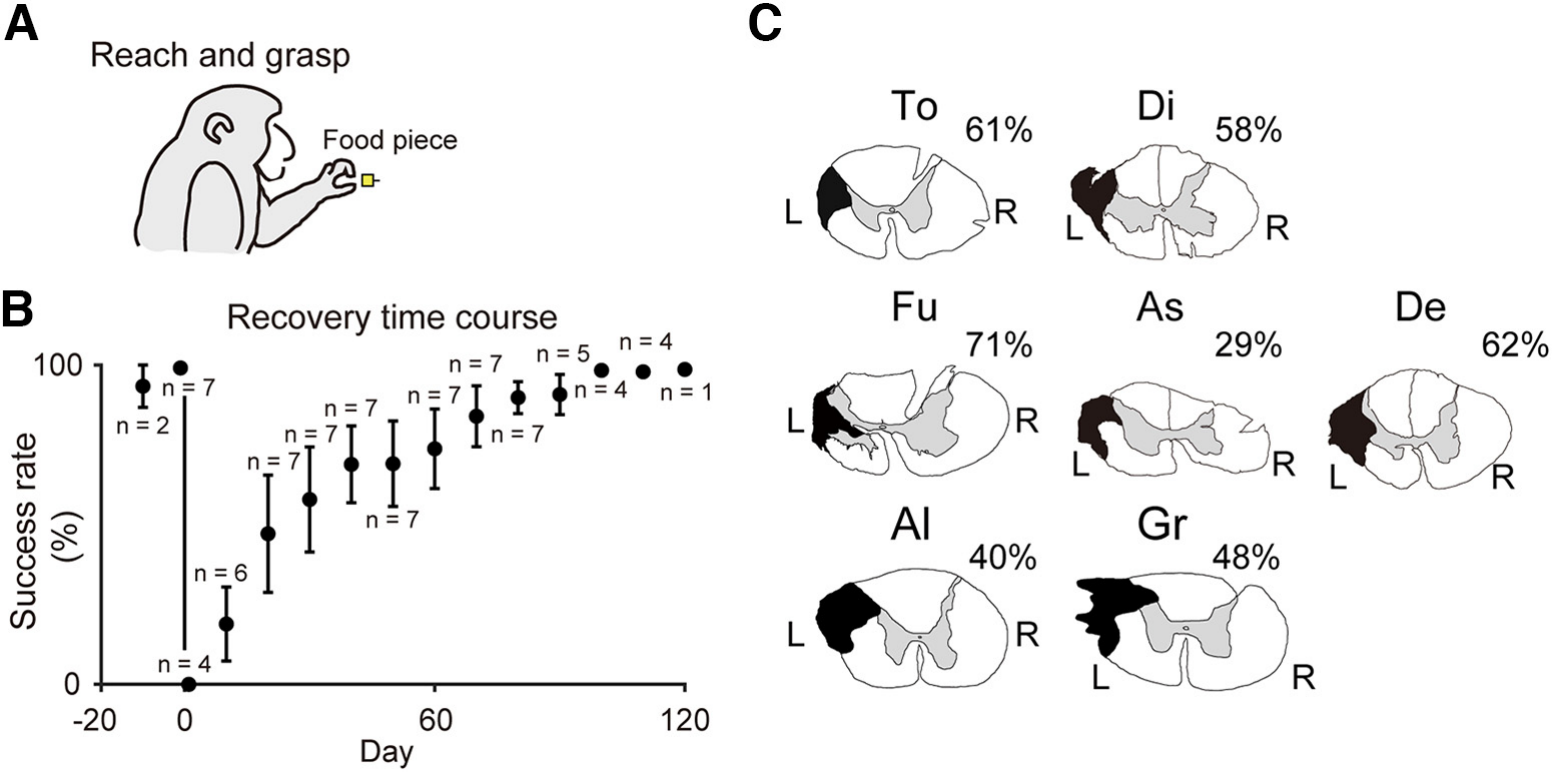
Recovery course of precision grip and spinal cord lesion. ***A***, Reach and grasping task (left). Representative image of Precision grip (right). ***B***, Recovery time course of the success rate of precision grip in seven monkeys. Each plot represents the mean success rate. Error bars indicate SEM; *n* means the number of monkeys on each day. A successful trial was defined as that in which the monkey succeeded in grasping the food morsel with precision grip using just the pads of the index finger and thumb and bringing it to the mouth to eat without dropping it. Data from the last behavioral test before the SCI (day −1 or day 0) are plotted on day 0. ***C***, The extent of spinal cord injury in each animal. The black shaded area indicates the maximum lesion extent of the spinal cord at the border of C4 and C5. The lesion extents of Monkeys Al and Gr were reconstructed from parasagittal sections of their spinal cord. The percentage value beside each panel shows the relative extent of spinal cord injury in each animal (see Materials and Methods). L: left, R: right.

Monkeys were trained to perform reach and grasp task with their left arms (Fig. 1*A*). They were trained to grasp pieces of sweet potato (5 × 5 × 5 mm) through a narrow slit (10 mm in width) on an acrylic panel placed in front of the home cage (Monkeys Di, Al, and Gr) or in front of the monkey chair on which they were seated (Monkeys To, De, As, and Fu; Fig. 1*A*). To evaluate the recovery course of finger dexterity, hand movements were filmed during task performance from the radial side (30 frames per second; shutter speed, 1/250 s). A “successful precision grip” was defined as the monkey grasping the piece of food using only the ventral sides of the index finger and thumb (Fig. 1*A*). The success rate of precision grip in the 30 trials from beginning of each daily session was calculated throughout the experimental period (Fig. 1*B*).

### Surgeries

All surgical procedures were performed under sterilized conditions. The monkeys were sedated by intramuscular injection of ketamine (Daiichi-Sankyo; 10 mg/kg, i.m.) plus xylazine (Bayer; 1 mg/kg, i.m.). Atropine (Tanabe Pharma; 0.01 mg/kg, i.m.), ampicillin (Meiji Pharma; 40 mg/kg, i.m.) and dexamethasone (Banyu Pharma; 0.25 mg/kg) were injected intramuscularly as premedication. During the surgery, anesthesia was maintained with intravenous injection of sodium pentobarbital (Abbott Laboratories; 25 mg/kg, i.v.) or isoflurane (MSD Animal Health; 1–2%) inhalation. ECG, SpO2, end-expiratory carbon dioxide pressure, and body temperature were monitored carefully during the surgery. Drips of Ringer’s solution were given throughout surgery. Dexamethasone (NICHI-IKO; 0.01 mg/kg, i.m.), ampicillin (Fujifilm Wako Chemical Corp.; 40 mg/kg, i.m.), and ketoprofen (Kissei Pharmaceutical Co, Ltd.; 2.0 mg/kg, i.m.) were administered after surgery.

#### Surgery 1: lesion of the corticospinal tract (CST)

The DLF, where the majority of CST and rubrospinal tract axons course, was transected at the border between the C4 and C5 segments on the left side of the spinal cord, as described previously (Sasaki et al., 2004; Nishimura et al., 2007; Sugiyama et al., 2013; Fig. 1*C*). A horizontal strip oriented in a mediolateral direction relative to the DLF was made by inserting a blunt L-shaped hook with a maximum insertion of 5 mm, which corresponds to the distance from lateral convexity of the spinal cord to the lateral edge of gray matter. Then, by using fine forceps, the dorsal part of the DLF was transected from the dorsal root entry zone ventrally to the level of the horizontal strip described above. Finally, the lesion was extended ventrally to the most lateral part of the lateral funiculus using forceps. The opening of the dura mater was closed, and the skin and back muscles were sutured with nylon or silk threads.

#### Surgery 2: neural tracer injection into the motor cortex

**Figure 2.**
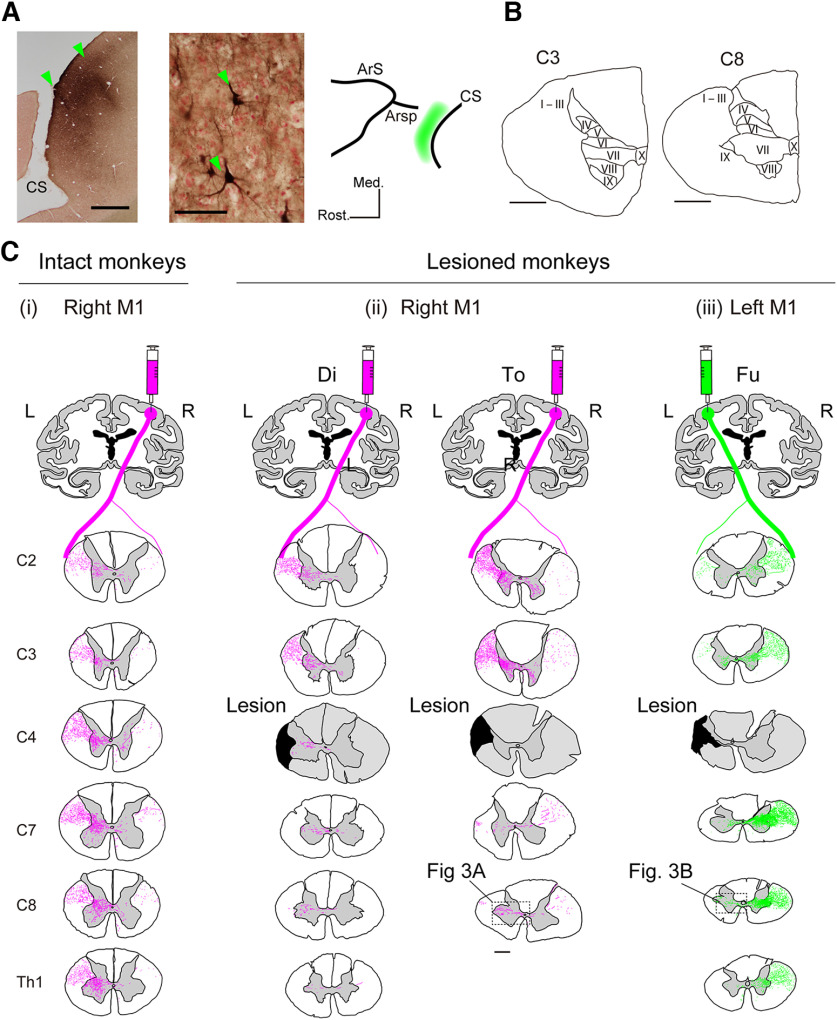
Injection sites of BDA and distribution of the BDA-labeled axons at different segments from C2 to Th1. ***A***, Left, Example of injection sites in M1; bulk of BDA-reaction product at the injection site in the gray matter of the rostral bank of the central sulcus (CS). Center, A high-magnification view of BDA-labeled pyramidal neurons in Layer V at the injection site in Mo Fu. Scale bars in left and center panel indicate 2 mm and 50 μm, respectively. Right, Schematic distribution of injection tracks of BDA (green area). Detailed location of injection sites is shown in Table 1. Arc, arcuate sulcus; Arsp, arcuate sulcus spur. ***B***, Definition of the laminae of Rexed in the C3 and C8 spinal segments (Rexed, 1952). Scale bars = 1 mm. ***C***, Distribution of BDA-labeled axons in the C2, C3, C4, C7, C8, and Th1 spinal segments originating from the right M1 in the intact monkey [Mo-4 from Yoshino-Saito et al., 2010; C(i)], and from the contralesional (right) M1 in Monkey To [C(ii)] and from the ipsilesional (left) M1 in Monkey Fu [C(iii)]. The black shaded area in the panels of C4 in C(ii) and C(iii) indicates the lesion extent at the border of C4 and C5 in these animals Scale bar = 1 mm.

After recording the behavioral data after the CST lesion (Di, 155 d; To, 563 d; Al, 278 d; Gr, 277 d; De, 107 d; As, 124 d; Fu, 731 d) anterograde neural tracer was injected into the primary motor cortex (M1). Under deep anesthesia with isoflurane, the animal was mounted on a stereotaxic frame. Craniotomy was performed to expose the hand area of M1 on the right (contralesional) side (Di, To, Al, and Gr) or left (ipsilesional) side (De, As, and Fu; Table 1; Fig. 2*A*,*C*). Cortical surface was exposed after opening the dura. As we described previously (Yoshino-Saito et al., 2010), 0.5-μl solution of biotinylated dextran amine [BDA; Invitrogen; 10,000 MW; 10% dissolved in 0.01% phosphate buffer (pH7.3)] was injected at each injection site in the hand area of M1 using a 10-μl Hamilton microsyringe. One to three injections were made in each track and totally 6–8 tracks were injected with BDA in each animal (details are shown in Table 1).

According to our previous study (Nishimura et al., 2007), the locations of the digit region of M1, as indicated by twitches of the digits and wrist induced by train stimuli of 15 pulses (biphasic pulses of 0.1 ms cathodal and 0.1 ms anodal, at 333 Hz) with the threshold below 40 μA (often below 10 μA) under sedation with ketamine (Daiichi-Sankyo; 10 mg/kg, i.m.), were distributed between 11 and 17 mm lateral from the midline and 1–2 mm anterior to the central sulcus (CS; Fig. 2*A*, right panel, green shaded area). The locations of the injection tracks were determined by this information. Injection tracks were in two rows, one was 1 mm anterior to the CS and the other was 2–3 mm anterior to the CS. The former tracks were located along the bank of the CS, which corresponds to the “new M1,” and the latter tracks were located on the convexity of the cortical surface, which correspond to the “old M1,” according to Rathelot and Strick (2009). The injection tracks in each row were separated by 2–3 mm medio-laterally.

### Histologic processing

One to six months after the injection of BDA (Di, 75 d; To, 114 d; Al, 93 d; Gr, 178 d; De, 42 d; As, 30 d; Fu, 139 d), monkeys were anesthetized deeply with an overdose of sodium pentobarbital (50–100 mg/kg, i.v.) and perfused transcardially with 0.1 m PBS (pH 7.3) containing heparin (1 unit/ml), followed by 4% paraformaldehyde in 0.1 m PBS (pH 7.3). Therefore, the total survival periods of the monkeys after the spinal cord injury were 230 d for Di, 677 d for To, 371 d for Al, 455 d for Gr, 149 d for De, 154 d for As, and 870 d for Fu (Table 1). After perfusion, the brain and spinal cord were removed and postfixed in the same fresh PBS containing 2% paraformaldehyde, followed by 10%, 20%, and 30% sucrose. After saturation with sucrose, a series of 50-μm-thick sections of the brain, including the injection sites, and cervical and thoracic spinal cord from C2 to Th2 segments were obtained using a freezing microtome (Thermo Scientific). The spinal segments were determined by identifying the level of dorsal and ventral rootlets. In five monkeys (Di, To, Fu, De, and As), the spinal cord was sectioned coronally, while in two monkeys (Al and Gr), the spinal cord was sectioned longitudinally. After BDA-labeling, the sections were mounted on gelatin-coated glass slides. The sections were collected serially in 0.1 m PBS, pH 7.4. Some sections were Nissl-stained with 1% Cresyl Violet to estimate the relative extent of the spinal cord injury. Following the methods described previously (Sugiyama et al., 2013), the extent of lesion (percent) in the DLF was determined by subtracting the ratio of remaining area of the lateral and ventral funiculi on the lesioned side divided by the whole area of the lateral and ventral funiculi from 100%.

In order to visualize the BDA labeling, the remaining sections were processed using the avidin-biotin-peroxidase method (Vector) with 3,3’-diaminobenzidine (DAB; Sigma) as the chromogen. Sections were washed twice in 0.05 m PBS for 10 min, followed by incubation with 0.6% hydrogen peroxide in methanol for 30 min to reduce the activity of endogenous peroxidase. After reduction, sections were washed three times in PBS, and incubated for 120 min in a solution containing 50-fold-diluted ABC in 0.05 m PBS plus 0.4% Triton X-100. The sections were rinsed twice in PBS and twice in 0.05 m Tris-buffered saline. The peroxidase was reacted and visualized by incubating the sections in a solution containing 0.01% DAB, 1.0% nickel ammonium sulfate, and 0.0003% hydrogen peroxide in 0.05 m Tris buffer, for 10–15 min at room temperature. The reaction was terminated by rinsing in Tris buffer followed by two rinses in PBS. After the DAB-nickel staining, the sections were mounted on gelatin-coated glass slides, some of which were lightly counterstained with 1% Cresyl Violet, dehydrated, and coverslipped.

### Analysis in coronal sections

We chose the C3 and C8 segments for quantitative analysis of the CST axons and terminals as the examples of segments rostral and caudal to the lesion, respectively, because the C3 segment contains a large fraction of the cell bodies of PNs, which presumably mediate the CST inputs to motoneurons, and the C8 segment contains the spinal motoneurons innervating the hand muscle and their premotor interneurons. Therefore, we expected to observe plastic changes in the CST at these segments. In order to quantify the number of axons and terminal bouton-like swellings, three sections separated by 400-μm intervals were selected from each segment. Detailed trajectories of BDA-labeled axons and their terminals were traced along with a section outline at 10× magnification, using a camera lucida attached to a light microscope (Nikon).

We traced and counted the number of BDA-labeled axons separately in the white matter including the dorsolateral, ventromedial, and dorsal funiculi on both sides to the BDA injections in the M1. All the axons, including those that ran in the longitudinal and transverse direction relative to the rostrocaudal axis of the spinal cord, were counted. We defined axons whose length was <150 μm as longitudinal axons, and those whose length was >150 μm as traverse axons in the coronal section, respectively, similar to our previous study (Yoshino-Saito et al., 2010). To evaluate the number of collateral branches rostral to the lesion, we divided the number of transverse axons (>150 μm) by the total number of axons (including longitudinal and transverse axons) in the white matter of bilateral DLF of the C3 segment. We compared these “transverse ratios” of the lesioned monkeys with those of the intact monkeys.

The number of axons labeled with BDA was counted in each lamina of the gray matter, regardless of the length included in each section, on both the ipsilateral and contralateral sides to the BDA injections. The border of each lamina was defined based on the cytoarchitecture according to the definition by Rexed (Rexed, 1952; Fig. 2*B*). When the same axon crossed the border between two laminae, the axon was counted as being included in both laminae. The number of BDA-labeled bouton-like swellings were also counted in each lamina in the C3 and C8 segments on both the ipsilateral and contralateral sides to the BDA injections. Then, we divided the number of labeled axons and bouton-like swellings in each lamina by the total number of the axons in the bilateral C3 DLF at each spinal segment for normalization, considering individual differences of the total number of labeled axons. We compared these values of lesioned monkeys with those of intact monkeys to evaluate the difference in the axonal and terminal distribution between these two groups.

### Analysis in longitudinal sections

In the longitudinal sections, we also evaluated the frequency of collateral branches in the C3 segment as representing the segments rostral to the lesion. At the C3 segment, we counted the number of labeled axons that crossed the border between the white matter and gray matter transversely. Then, we calculated “transverse ratios” similarly to the coronal sections.

To evaluate the frequency of the collateral projections that descended in the gray matter caudal to the lesion, we counted the number of labeled axons within 5 mm caudally from the level of the spinal lesioning. We also measured the direction of axons against the orientation of the central canal, and the length of axons in three longitudinal sections. They were then compared between intact and lesioned monkeys. Labeled axons were traced by Illustrator 25.2.3 (Adobe) and saved in a Scalable Vector Graphics file. Then, starting and ending points of the traced axons were determined using the original MATLAB script (The MathWorks). The length of axons was defined as the length between the starting and ending points, and the angle of axons was defined as the angle of the line that connects these two points of the axon against the orientation of the central canal. The angle ranges from 0° to 90°. The relationship of the length and angle was plotted. Based on the scatter plot, a two-dimensional histogram was created for the length and angle of axons, where each bin was normalized by the bin of the axons with the shortest length and highest angle. We made the same measurements in the corresponding segments of intact monkeys and compared the results with those of the monkeys with SCI.

### Experimental design and statistical analyses

Statistical analysis was performed using MATLAB. Wilcoxon test was performed to compare the percentage of axons and bouton-like swelling between lesioned and intact monkeys. Bonferroni correction was used for seven times of repeated measurements of each laminae component (*p* = 0.05/7); *p*-values are provided in each figure.

## Results

### Lesion extent

Figure 1*C* shows the extent of the C4/C5 DLF lesion in the seven monkeys. The lesions in the monkeys were intended to encompass the normal distribution of the l-CST. The lesion extent ranged from 29% to 71% of the lateral and ventral funiculi, which mostly corresponds with the lesion extent reported previously (Nishimura et al., 2007, 2009; Sugiyama et al., 2013; Sawada et al., 2015; Chao et al., 2019). Lesion extent was regarded as covering at least the majority of the l-CST axons in all monkeys.

### Recovery time course

The success ratio of the precision grip task was nearly 100% before the lesion, but dropped to 0% immediately after the SCI. As shown in Figure 1*B*, however, the success ratio gradually recovered and reached 100% within three months in all seven monkeys (Fig. 1*B*), and remained constant afterward, similar to the results in our previous studies (Nishimura et al., 2007, 2009; Sugiyama et al., 2013; Sawada et al., 2015; Chao et al., 2019).

### Overview of the CST originating from the contralesional (right) and ipsilesional (left) M1

We investigated the trajectories of the CST axons from the contralesional (*n* = 4) and ipsilesional (*n* = 3) hand area of M1 using an anterograde neural tracer, BDA (Fig. 2). Figure 2*A*, left, shows a section along the rostral bank of the central sulcus. As described in Materials and Methods, multiple injections were made along these tracks (Fig. 2*A*, right). As shown in this figure, the injection of BDA was successful in labeling cortical neurons located at various depths in the precentral gyrus. The labeled neurons around the injection tracks included large pyramidal neurons, which may include CST neurons in Lamina V of M1 (Fig. 2*A*, middle).

Figure 2*C(i)* shows the trajectories of CST axons in an intact animal (Mo-4), taken from our previous study (Yoshino-Saito et al., 2010). As described previously, the majority of CST axons (85–98%) descend in the DLF on the contralateral side (left, L) to the injection (right, R) and issue collaterals to various segments in the cervical spinal cord (Kuypers, 1982; He et al., 1993; Armand et al., 1997; Rosenzweig et al., 2009). In the upper–middle cervical segments (C2, C3, C4), these collaterals terminate mainly in the contralateral Laminae VI and VII (compare Fig. 2*B*) and partly to the ipsilateral Lamina VIII after recrossing the midline. A small number of CST axons descended in the ipsilateral DLF. Some collaterals appeared to enter the gray matter (see the C4 segment) and terminate in Laminae VI, VII, and VIII of the ipsilateral side, but it was difficult to follow the axonal trajectory completely because they were mixed with the recrossing axons from those descending in the contralateral side. In the lower cervical segments (C7, C8, Th1), in addition to the terminals in Laminae VI and VII, the axons reached Lamina IX on the contralateral side. Some of these axons appeared to be traced to the ipsilateral Lamina VIII after recrossing. A small number of collaterals from the ipsilateral, uncrossed CST in the ipsilateral DLF were rarely traced to the gray matter [Fig. 2*C(i)*].

Below. we describe the results from animals with a variety of survival time after the spinal cord injury. However, basically similar results were observed between the animals with long postinjection and postinjury survival times and those with short postinjection and postinjury survival times. Therefore, we did not differentiate them depending on the survival time.

#### CST originating from the contralesional (right) M1

**Figure 3.**
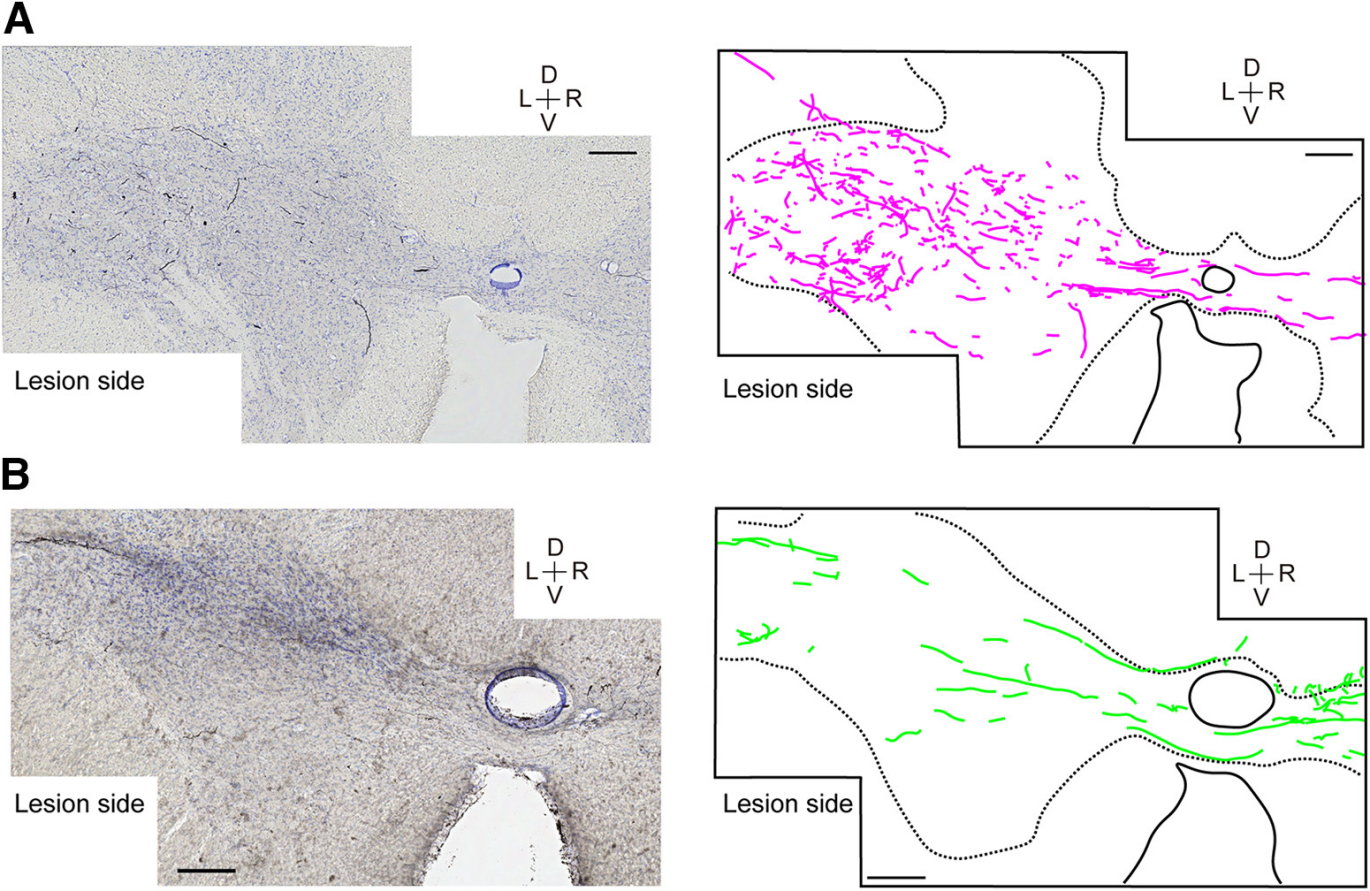
***A***, CST axons descending from the contralesional M1 in Monkey To at the C8 segment [compare Fig. 2*C(ii)*]. Photomicrograph in the left and camera lucida tracing of the BDA-labeled axons (in magenta) in the right panel. D, dorsal; V, ventral; L, left; R, right. Scale bar = 200 μm. ***B***, CST axons descending from the ipsilesional M1 in Monkey Fu at C8 segment [compare Fig. 2*C(iii)*]. Photomicrograph in the left and camera lucida tracing of the BDA-labeled axons (in green) in the right panel. Scale bar = 200 μm. Note that the CST axons in ***A*** are mostly short and penetrate the section perpendicularly, while those in ***B*** are traversing the section horizontally for a long distance.

Figure 2*C(ii)* shows examples of the axonal trajectories of the CST originating from the contralesional (right) M1 in the lesioned animals [Monkeys Di (230 d after SCI) and To (677 d after SCI)]. Few labeled axons were observed immediately caudal to the lesion in the ipsilesional DLF in these contralesional-injection group, which suggests that some CST axons remained uncut, but the regrowth of axons penetrating the scar of lesion was minimal, if any. In both monkeys, rostral to the C4/C5 lesion (C2, C3), a number of collaterals issued from the main axons into the left DLF to the gray matter. The majority terminated in Laminae VI/VII in the left spinal gray matter, but some crossed the midline and terminated mainly in Lamina VIII on the contralesional side. The majority of descending axons in the DLF were transected by the lesion at C4/C5. However, caudal to the lesion (C7, C8), some axons could be observed in Laminae VII and IX on the ipsilesional side [C8 in Fig. 2*C(ii)*]. As shown in the photomicrograph and its camera lucid drawing in Figure 3*A*, these axons were mainly penetrating the section perpendicularly because their horizontal length in the section was shorter than 150 μm. The origin of these axons will be discussed in the later section.

#### CST originating from the ipsilesional (left) M1

Figure 2*C(iii)* shows an example of the axonal trajectories of the CST originating from the ipsilesional (left) M1 in the lesioned animal [Monkey Fu (870 d after SCI)]. Rostral to the C4/C5 lesion (C2, C3), the CST axons were mainly located in the contralesional DLF. A number of collaterals issued to Laminae VI and VII in the gray matter on the contralesional side at the C3, some of which further crossed the midline and terminated in Lamina VIII of the ipsilesional side. Caudal to the lesion (C7, C8, Th1), a large number of collaterals issued from the main axons into the DLF to the gray matter and terminated in Laminae VI and VII on the right side. As shown in the photomicrograph and its camera lucid drawing in Figure 3*B*, a majority of these collaterals crossed the midline, traversed the gray matter, and terminated in the ventral horn including Lamina IX on the ipsilesional (left) side of the spinal cord.

Table 2 shows the number of BDA-labeled axons in the DLF at the C3 and C8 segments on the ipsilesional and contralesional sides. As shown in the table, the BDA-labeled axons were markedly reduced at the C8 segment in the ipsilesional DLF in the lesioned monkeys after BDA injection in both the contralesional (Di and To) and ipsilesional M1 (Fu, De, As). These results suggest that the transection of CST axons was mostly perfect. Conversely, there was only slight decrease in the number of axons (Mo-2, Mo-3, Mo-4) or even increase (Mo-1) in the number of BDA-labeled axons at the C8 segment compared with the C3 in the intact monkeys. The increase might reflect the branching of the CST axons in the white matter before innervating the gray matter of the lower cervical segments.

**Table 2.** Mean ± SD of the number of labeled axons in the coronal slices in each component of the white matter at C3 and C8 in individual monkeys with coronal sections

Name	Injected side	Segment	DLF right	Left
Di	Right	C3	1.7 ± 2.0	339.3 ± 22.7
		C8	0.3 ± 0.5	2.0 ± 2.6
To	Right	C3	94.3 ± 2.3	1169.0 ± 54.2
		C8	41.3 ± 8.3	15.7 ± 4.5
De	Left	C3	280.7 ± 17.8	24.3 ± 2.9
		C8	290.0 ± 6.2	0
As	Left	C3	341.7 ± 9.1	43.3 ± 4.0
		C8	412.3 ± 11.7	2.0 ± 3.5
Fu	Left	C3	470.7 ± 54.2	33.0 ± 8.0
		C8	234.7 ± 43.6	0
Mo1	Right	C3	3.3 ± 18.9	335.5 ± 18.9
		C8	2.0 ± 1.0	414.0 ± 6.6
Mo2	Right	C3	22.0 ± 3.6	213.3 ± 9.1
		C8	20.7 ± 7.6	207.7 ± 33.1
Mo3	Right	C3	40.0 ± 10.4	451.0 ± 61.7
		C8	38.0 ± 1.7	377.0 ± 10.5
Mo4	Right	C3	68.7 ± 5.8	467.3 ± 55.6
		C8	56.3 ± 13.6	466.7 ± 34.1

The data from the intact animals were obtained from our previous study (Mo-1 to Mo-4 in Yoshino-Saito et al., 2010).

### The number of CST axon collaterals entering the gray matter

**Figure 4.**
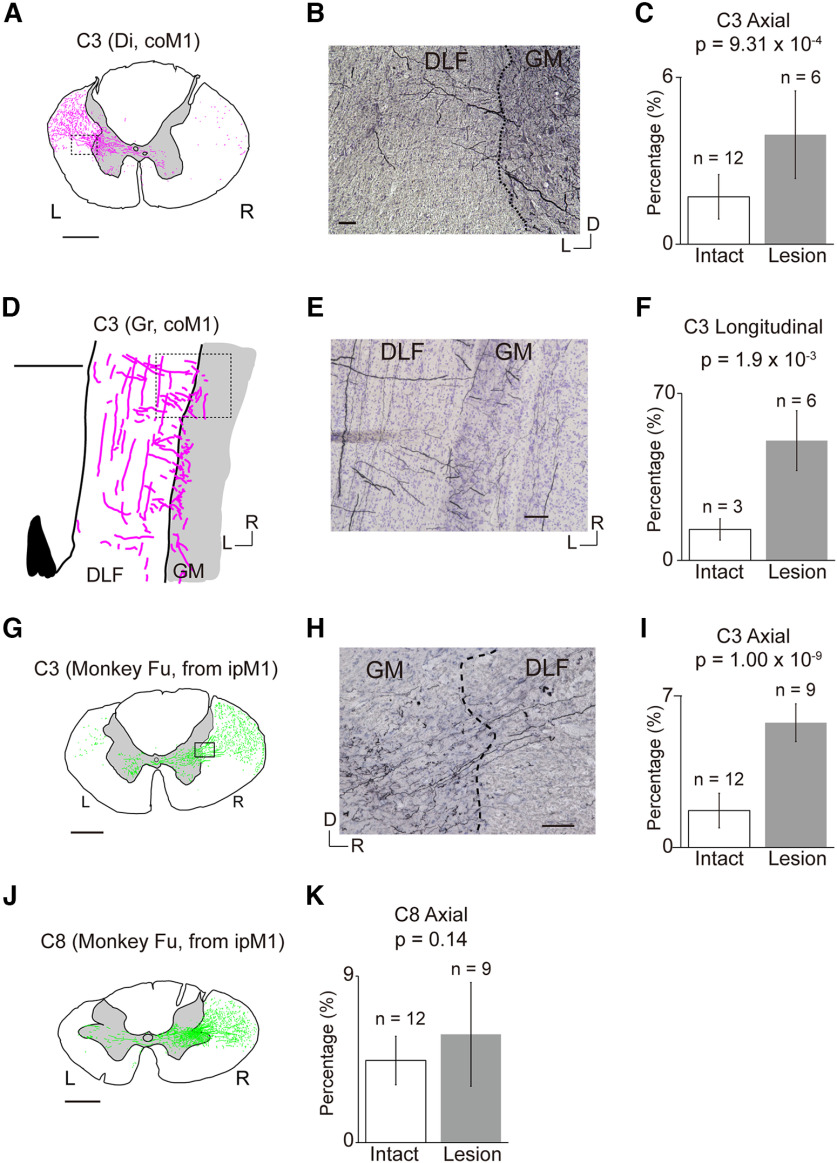
Collateral branching from CST axons originating from the contralesional M1 (coM1) or ipsilesional M1 (ipM1) at the C3 (***A–I***) and C8 segment (***J***, ***K***). ***A***, An example trace of CST axons in an axial section showing transverse CST axons entering the gray matter from the ipsilesional dorsolateral funiculus (DLF) at C3 in a monkey with BDA injection into the coM1 [Monkey To, the same as Fig.2*C(ii)*, C3]. R, right; L, left. Scale bar = 1 mm. ***B***, A high magnification view of the square in ***A***. The dashed line indicates the border between the gray matter and white matter (DLF). D, dorsal; L, left. Scale bar = 50 μm. ***C***, Bar graphs indicate the percentage of transverse axons among the labeled axons in ipsilesional DLF at C3. Data were obtained by averaging the number from three axial sections from each monkey. Error bars indicate standard deviation. Intact monkeys (Mo-1, Mo-2, Mo-3, Mo-4); lesioned monkeys (Monkeys To and Di). ***D***, An example trace of longitudinal section of the C3 segment showing transverse axons (black arrow) entering the gray matter from the ipsilesional DLF at C3 in a monkey with BDA injection into the coM1 (Monkey Gr). Descending axons are indicated with black arrowheads. A dashed line indicates the border between gray matter and white matter. R, rostral; L, left. Scale bar = 1 mm. ***E***, High-magnification view of the square in ***D***. The dashed line indicates the border between gray matter and white matter. R, rostral; L, left. Scale bar = 100 μm. ***F***, Percentage of transverse axons among the labeled axons in contralesional DLF at C3 in monkeys with BDA injection into the coM1 (Monkeys Al and Gr). ***G***, An example trace of CST axons in an axial section showing transverse CST axons entering the gray matter from the contralesional DLF at C3 in the monkey with BDA injection into the ipM1 [Monkey Fu, the same as Fig.2*C(iii)*, C3]. Scale bar = 1 mm. ***H***, High magnification of the square in ***G***. D, dorsal; R, right. Scale bar = 100 μm. ***I***, The percentage of transverse axons out of the total number of labeled axons in the contralesional DLF at C3. Data were obtained from the axial sections of Monkeys Fu, As, and De. ***J***, An example trace of CST axons in an axial section showing transverse CST axons entering the gray matter from the contralesional DLF at C8 in a monkey with BDA injection into the ipsilesional M1 [Monkey Fu, the same as Fig.2*C(iii)*, C8]. Scale bar = 1 mm. ***K***, Percentage of transverse axons among the labeled axons in contralesional DLF at C8 in monkeys with BDA injection into the ipM1 (Monkeys De, As, and Fu).

Figure 4*A*,*B* shows transverse axons entering the gray matter (GM) in a coronal section of the C3 segment from the ipsilesional DLF in monkeys with BDA injection into the contralesional M1. The number of transverse axons among the total number of axons descending in the DLF at the C3 segment from the contralesional M1 was significantly larger in the lesioned monkeys than in the intact monkeys (Fig. 4*C*). Figure 4*D*,*E* shows transverse axons entering the GM from the ipsilesional DLF in a longitudinal section. The number of transverse axons among the total number of axons descending in the ipsilesional DLF at the C3 segment was significantly larger in the lesioned monkeys than in the intact monkeys (Fig. 4*F*). Conversely, Figure 4*G*,*H* shows transverse axons entering the gray matter of the C3 segment from the contralesional DLF in coronal sections of the monkeys with BDA injection into the ipsilesional M1. The number of transverse axons among the total number of axons descending in the contralesional DLF at the C3 segment was significantly larger in these lesioned monkeys than in intact monkeys (Fig. 4*I*). Transverse axons from the ipsilesional M1 tended to increase at the C8 segment, but the difference was not significant (Fig. 4*J*,*K*).

**Figure 5.**
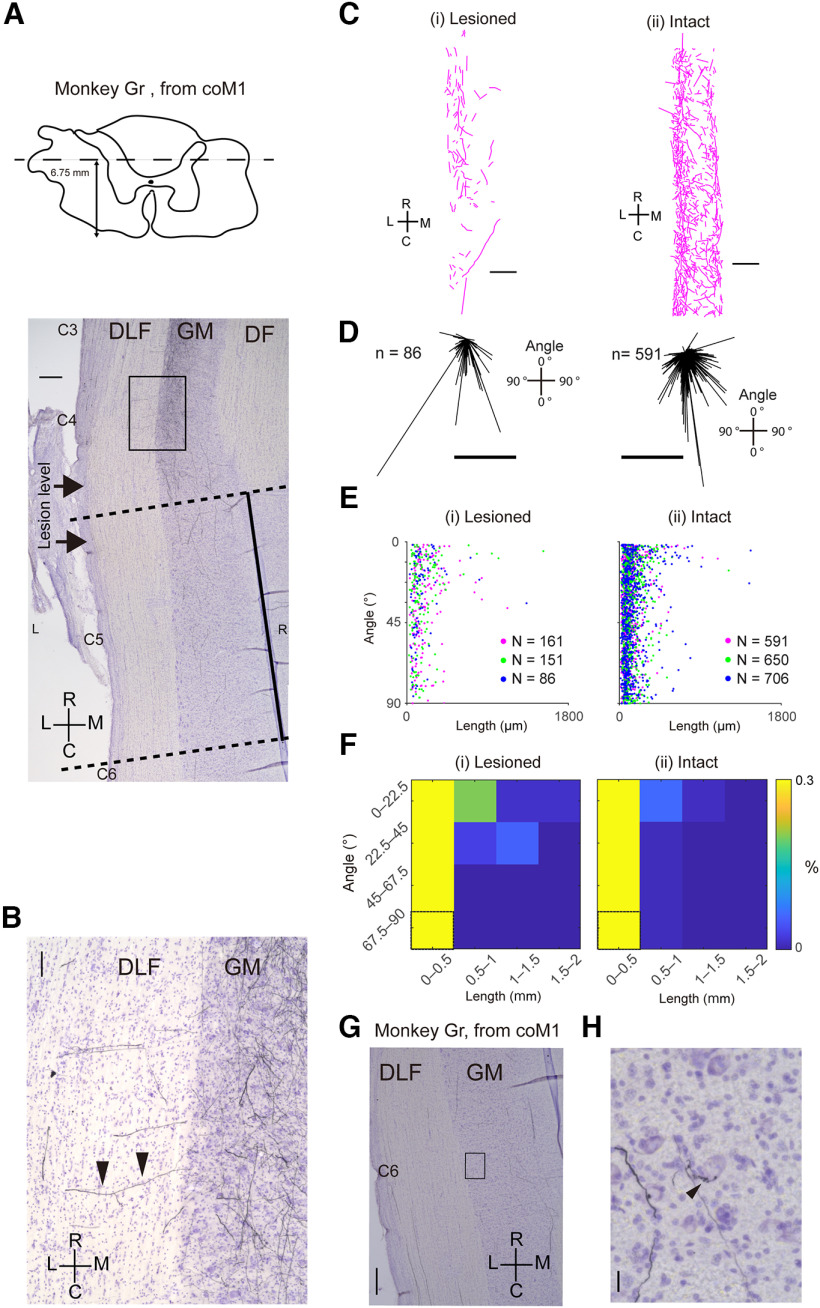
Longitudinal sections of the cervical spinal cord showing CST axons from the contralesional M1 (coM1) that descended in the ipsilesional gray matter. ***A***, Upper panel, The slice obtained at 6.75 mm dorsal from the ventral end of the spinal cord sections (dashed line). Lower panel, A photomicrograph of a longitudinal section spanning C3–C6 segments showing the BDA-labeled CST axons. Horizontal arrows at C4–C5 indicate the levels of rostral and caudal borders of the C4/C5 lesion. The black square corresponds to the area of ***B***. The dashed line indicates the rostral and caudal ends of **C**. The black line indicates the direction of the central canal, which corresponds to the R-C axis in ***C***. Scale bar = 500 μm. ***B***, A high magnification view of the square in ***A***. Arrowheads show a BDA-labeled CST axon that ran horizontally and entered the gray matter from the DLF. Scale bar = 100 μm. ***C***, Traced axons in the gray matter at the C5 segment of a lesioned monkey [(i); Monkey Gr] and intact monkey [(ii); Mo-5]. Scale bars = 500 μm. ***D***, Direction (see the inset) and length of individual axons in ***C(i)*** and ***C(ii)*** are indicated with the distribution of vectors in the left and right panels, respectively. Scale bar = 50 μm. ***E***, Relationship between length of each axon piece and its direction shown in ***D*** in three sections from Monkey Gr (i) and Mo5 (ii; different color assignment), respectively. ***F***, Density map of the axon pieces with different length (horizontal axis) and different direction (vertical axis). Data obtained from ***E***. The values were normalized by the overall number of the labeled axons in each condition. Encased bins indicate the baseline for the normalization in each panel. ***G***, Photomicrograph of the C6 segment. Magnified view of the square is indicated in ***H***, where the axons are terminated on a large, presumptive motoneuron. The black arrowhead indicates a presumptive bouton-like swelling terminating on the motoneuron. R, rostral; C, caudal; L, lateral; M, medial; DLF, dorsolateral funiculus; GM, gray matter; DF, dorsal funiculus. Scale bars in ***G*** and ***H*** indicate 500 and 20 μm, respectively.

### Axons descending in the gray matter bypassing the DLF lesion

In Figures 2*C(ii)* and 3*A*, we described the existence of CST axons in the gray matter of the ipsilesional C8 segments originating from the contralesional M1. In the coronal sections obtained from Monkey To, we observed some axons that appeared to descend through the gray matter (GM) at the level of lesion (C4) and continued to descend in the gray matter to reach the C8 segments. We confirmed such descending axons in the longitudinal sections obtained from Monkey Gr (Fig. 5). Figure 5*A* shows the overview of a sagittal section spanning from the C3 to C6 segment. Rostral to the lesion (the level between the two horizontal arrows in C4–C5), a number of descending axons in the white matter were sending collaterals to the gray matter (Fig. 5*B*, arrows), which terminated there. Many axons appeared to further run in the rostro-caudal direction in the GM. Figure 5*C* shows the stained CST axons in the GM caudal to the lesion (C5; between the two dotted lines in Fig. 5*A*) in Monkey Gr [Fig. 5*C(i)*] and in the corresponding area in the intact monkey [Fig. 5*C(ii)* from Mo-5 in Yoshino-Saito et al., 2010]. Figure 5*D(i)*,*(ii)* analyzed the direction (see the inset) and length of individual axon pieces in Figure 5*C(i)*,*(ii)*, respectively, and their distribution is shown in Figure 5*E(i)*,*(ii)* and their normalized values are shown in the density map of Figure 5*F(i)*,*(ii)*. The results suggest that axons running in the longitudinal direction (close to 0°) already existed in the intact state. However, the proportion of long axons running in the longitudinal direction [angle < 33.75° and length > 500 μm in Fig. 5*F(i)*, data from both Monkeys Al and Gr] appeared to have increased in the animals with SCI. Further, some of the longitudinal axons reached the C6/C7 segment and appeared to terminate on motoneuron-like large neurons (Fig. 5*G*,*H*). These results suggested the possibility that the number of CST axons descending in the gray matter for >1.5 mm appeared to have increased after SCI, some of which established connections with motoneurons in the lower cervical segments.

### Distribution of CST axons and terminal boutons originating from the contralesional (right) M1

**Figure 6.**
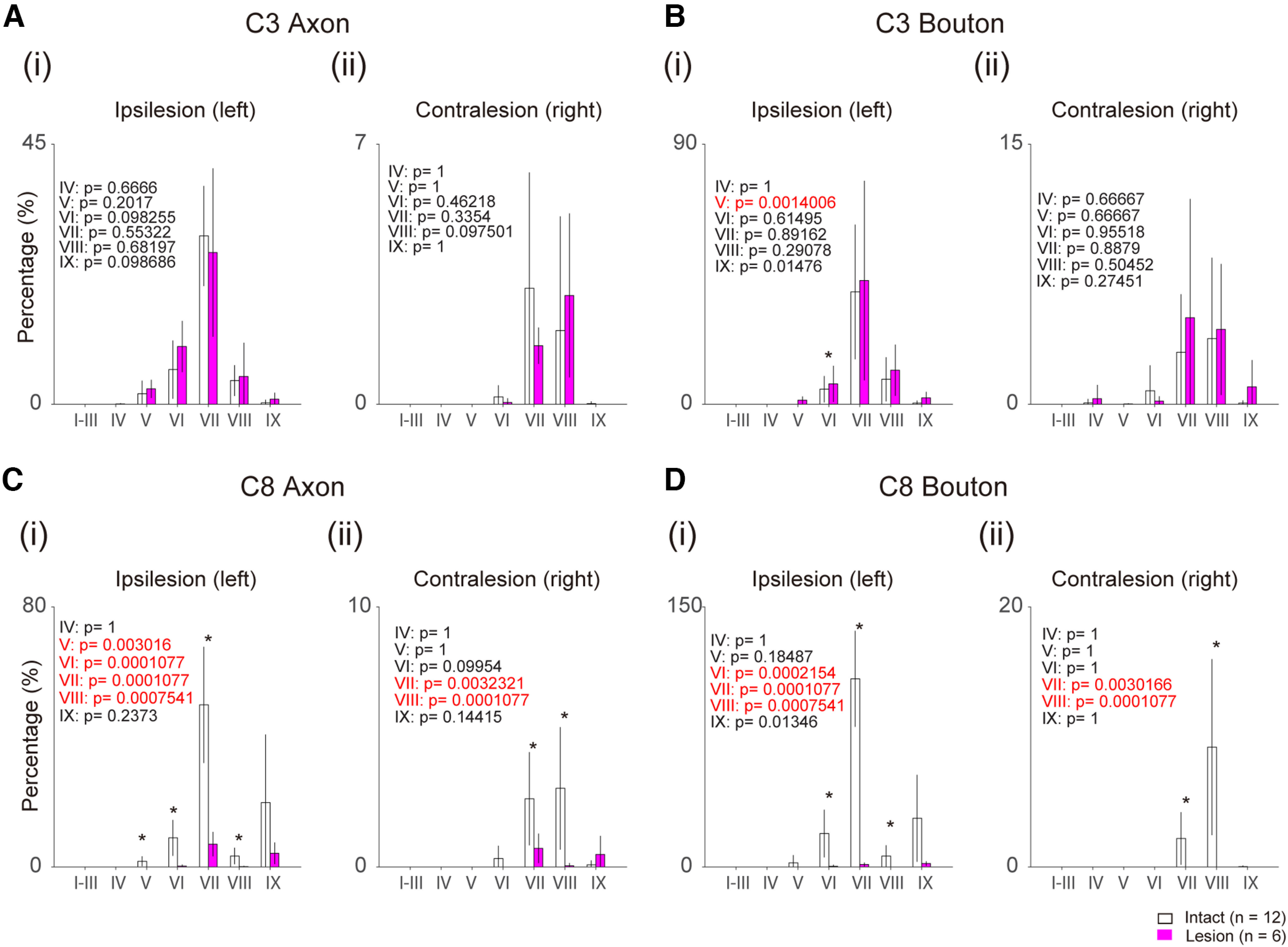
Comparison of the number of CST axons and boutons originating from the contralesional M1 between the intact (white columns) and lesioned (magenta columns) animals. ***A***, Histograms indicate the number of axons in each lamina of the ipsilesional (i) and contralesional C3 (ii), divided by the total number of the axons in the DLF on both sides at C3. Insets in each panel indicate the *p*-value of *t* test. ***B***, The number of bouton-like swellings in each lamina of the ipsilesional (i) and contralesional C3 (ii). ***C***, The number of axons in each lamina of the ipsilesional (i) and contralesional C8 (ii). ***D***, The number of bouton-like swellings in each lamina of the ipsilesional (i) and contralesional C8 (ii). *p < 0.05/7. Red letters in the insets indicate statistically significant values. Three sections are obtained from each monkey. The number of intact and lesioned monkey are four and two, respectively.

Figure 6 shows the relative distribution of axons and bouton-like swellings in each lamina (Fig. 2*B*) of the ipsilesional (left) and contralesional (right) C3 and C8 segments in the monkeys with BDA injections into the contralesional M1. For this quantitative analysis, we selected C3 and C8 as representing the segments above and below the lesion, respectively, because we found the similar observations in other segments as shown in Figure 2*C*. The values were normalized as the percentage of descending axons in the DLF at the C3 segment on both sides. Rostral to the lesion (C3), as shown in Figure 6*A*, there was no significant difference in the number of axons in each lamina in both the contralesional and ipsilesional sides between the intact and lesioned animals. As for the number of bouton-like swellings, there was subtle increase in the ipsilesional Lamina VI in the lesioned animals (Fig. 6*B*). Caudal to the lesion (C8), the number was drastically decreased because of the lesioning, however, some axons and boutons were confirmed in the Laminae VI/VII and IX on the ipsilesional side (Fig. 6*C*,*D*), as has been described in the preceding sections [Figs. 2*C(ii)*, 3*A*, 5*C*].

### Distribution of CST axons and terminal boutons originating from the ipsilesional (left) M1

**Figure 7.**
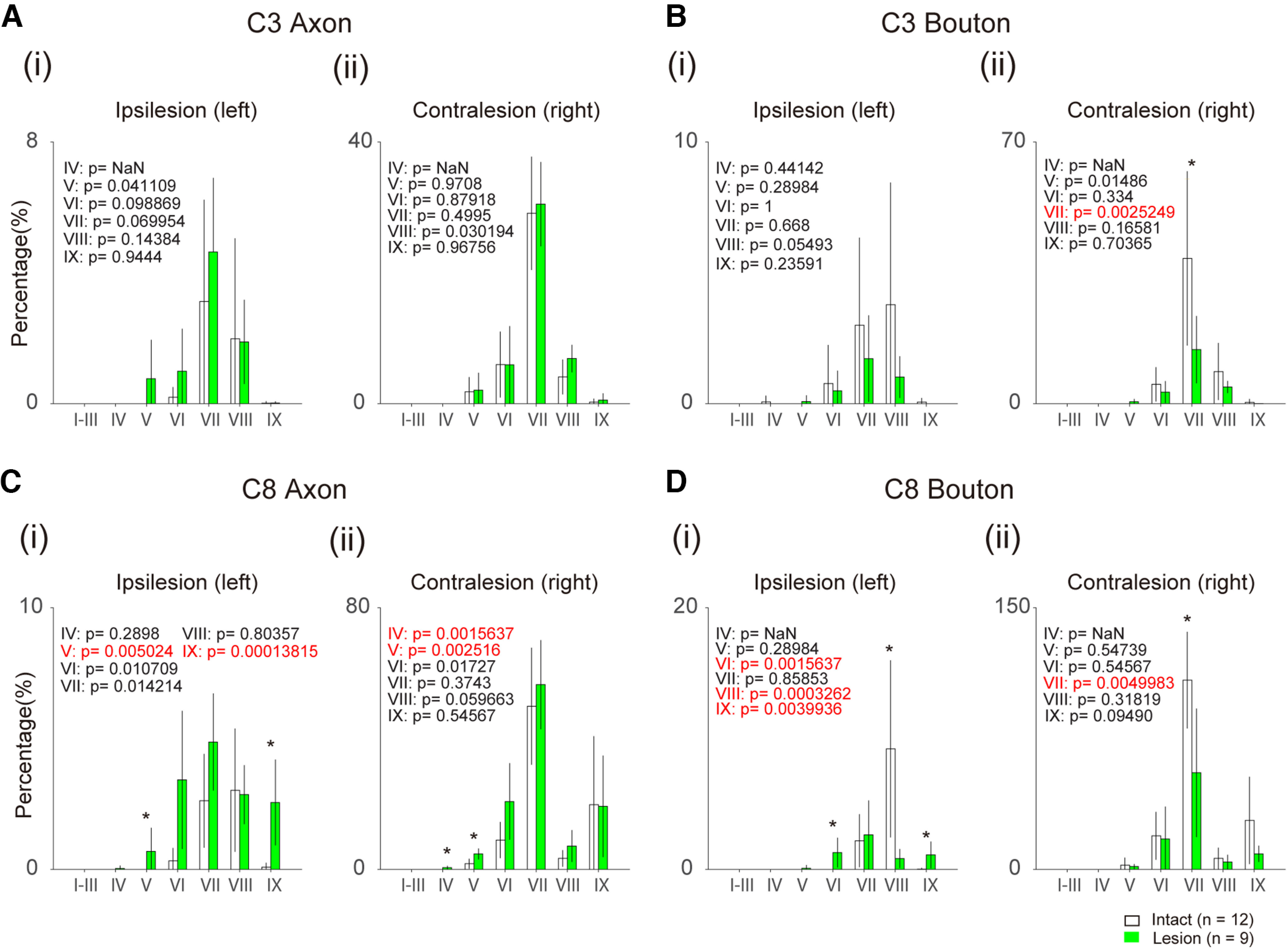
Comparison of the number of CST axons and boutons originating from the ipsilesional M1 between the intact (white columns) and lesioned (green columns) animals. ***A***, Histograms indicate the number of axons in each lamina of the ipsilesional (i) and contralesional C3 (ii), divided by the total number of the axons in the DLF on both sides at C3. Insets in each panel indicate the *p*-value of *t* test. ***B***, The number of bouton-like swellings in each lamina of the ipsilesional (i) and contralesional C3 (ii). ***C***, The number of axons in each lamina of the ipsilesional (i) and contralesional C8 (ii). ***D***, The number of bouton-like swellings in each lamina of the ipsilesional (i) and contralesional C8 (ii). *p < 0.05/7. Red letters in the insets indicate statistically significant values. Three sections are obtained from each monkey.

Figure 7 shows the relative distribution of axons and bouton-like swellings in each lamina (Fig. 2*B*) of the ipsilesional (left) and contralesional (right) C3 and C8 segments in the monkeys with BDA injections into the ipsilesional M1. The values were normalized as the percentage of descending axons in the DLF at the C3 segment on both sides. Rostral to the lesion (C3), as shown in Figure 7*A*, no significant difference was observed in the number of axons between the intact and lesioned animals. The number of bouton-like swellings decreased in the contralateral Lamina VII in the lesioned animals (Fig. 7*B*). Caudal to the lesion (C8), the number of axons increased in Laminae V and IX on the ipsilesional side and in Laminae IV and V on the contralesional side (Fig. 7*C*). The increase in the axons on the ipsilesional side should be mostly derived from the crossing axons from the contralesional DLF, as shown in Figure 3*B*. The number of bouton-like swellings also increased in Laminae VI and IX, but decreased in Lamina VIII on the ipsilesional side. Conversely, they also decreased in Laminae VII in the contralesional side. Implication of these findings will be addressed in Discussion.

## Discussion

**Figure 8.**
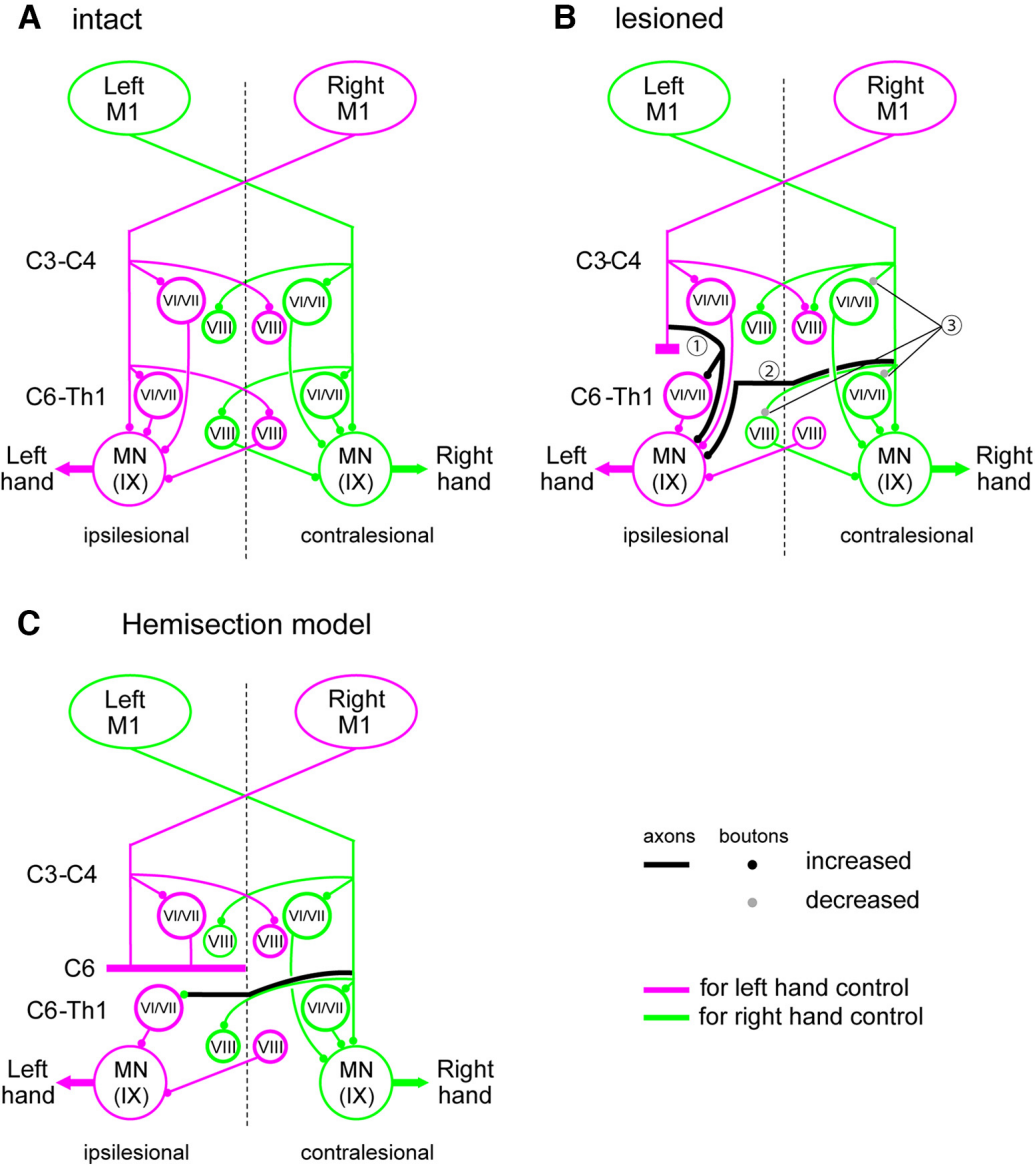
Schematic diagrams of the corticospinal projections originating from the forelimb area of M1 to individual spinal lamina of the C3–C4 and C6-Th1 segments in intact animals (***A***) and monkeys with the DLF lesion at C4/C5 (***B***). The increase and decrease of axons and boutons in Laminae I–V are not reflected in this schema because motor control is mainly related to Laminae VI–IX. The decrease of axons and boutons caudal to lesion from contralesional M1 are not reflected in this schema. They are compared with the observation of the hemisection models by Galea and Darian-Smith (1997) and Rosenzweig et al. (2010) which showed poorer recovery (***C***). The magenta and green indicates the projections from M1 for left-hand and right-hand control, respectively. ***A***, A schematic diagram of the projections of the CST projections from the bilateral M1 to the C3 and C8 segments summarized from our previous study (Yoshino-Saito et al., 2010). ***B***, ***C***, The same arrangement as ***A***. Black lines and circles indicate the increased axons and boutons, respectively. Dotted black lines and gray circles indicate decreased axons and boutons, respectively. Uncrossed CST axons are intentionally removed from the figures because their contribution to the hand movement control has not been clearly demonstrated so far.

In this study, we analyzed the trajectories of CST axons from both the contralesional and ipsilesional M1 in animals at 5–29 months after injury to clarify the neural pathways that support prominent recovery. Morphologic changes in CST from the bilateral M1 were detected at spinal segments both rostral and caudal to the lesion. These changes included regeneration of the direct corticomotoneural connection from the contralesional M1 and emergence of those from the ipsilesional M1. The results suggest that the corticospinal pathways from bilateral M1 may mediate cortical commands to control hand motoneurons. Figure 8 summarizes the corticospinal projections before the lesion (Fig. 8*A*; Yoshino-Saito et al., 2010) and after the full recovery (Fig. 8*B*). We further compared them with those reported previously on the hemisection models (Fig. 8*C*; Galea and Darian-Smith, 1997; Rosenzweig et al., 2010).

### Recovery mechanisms in the C4/C5 DLF lesion model

Previous studies have shown that the ability of dexterous hand movements can recover prominently in monkeys with DLF lesions that transected the l-CST and rubrospinal tract at the C4/C5 segment (Sasaki et al., 2004; Nishimura et al., 2007, 2009; Sugiyama et al., 2013; Sawada et al., 2015; Tohyama et al., 2017). In this model, selective blockade of C3–C4 PNs was shown to impair the recovering hand movements during the early recovery stage, but did not cause change during the late stage, suggesting involvement of other pathways in recovery (Tohyama et al., 2017). At the cortical level, reversible inactivation of the ipsilesional M1 impaired the recovering hand movements at the early stage, but did not in the late stage (Nishimura et al., 2007). These observations suggest that the long-term recovery is supported by different neural systems from the short-term recovery. As described above, in the short-term recovery, the residual descending pathways to the spinal interneurons including the C3–C4 PNs and their intersegmental connections to hand motoneurons would have been the major sources which compensated for the function of lesioned CST. In addition, the latent descending pathway from the ipsilesional M1 to motoneurons mediated by the reticulospinal neurons, which was clarified in cats by Jankowska et al. (2006), would be another source of early recovery. Thereafter, morphologic changes in the residual CST might have happened and been involved in the long-term recovery. In present study, we focused on the morphologic changes in corticospinal projections responsible for the long-term recovery (more than three to four months).

### CST projections from contralesional M1

Rostral to the lesion, collateral branches from the CST axons entering the gray matter of the C3 segment from both the contralesional and ipsilesional M1 were increased (Fig. 4*A–I*). However, no marked change was observed in the number of axons and bouton-like swellings in the gray matter (Figs. 6*A*,*B*, 7*A*,*B*). The increase of the collateral branches may suggest that the PNs in the C3 segment might contribute to recovery after the injury. However, it is difficult to conclude that the PNs are playing a major role in compensation of the impaired functions by the CST lesion because of the lack of increase in the axons and boutons in the C3 gray matter. This is consistent with our previous results, in which selective blockade of the PNs caused clear deficits during the early recovery but it was not the case long after the lesion (Tohyama et al., 2017). Caudal to the lesion (C8), axons (Fig. 6*C*) and bouton-like swellings (Fig. 6*D*) originating from the contralesional M1 decreased drastically by the lesion. However, some axons and terminals were observed in Laminae VI/VII and IX (Fig. 6*C*,*D*), which might have originated, at least in part, from recrossing of uncrossed CST axons descending in the contralesional DLF. However, our microscopic investigation suggests that the number of recrossing axons (Fig. 3*A*) was small and not large enough to explain the majority of axons and boutons. Instead, we confirmed that the majority of these axons were derived from the CST axons which originated from the ipsilesional DLF, projected to the gray matter rostral to the lesion, and descended in the gray matter bypassing the lesion (Figs. 3*A*, 5*F*, 8*B*, ①). Such axons were observed in both Monkeys Di (233 d after SCI) and To (677 d after SCI). Comparison with intact animals suggested that some of these descending axons that bypassed the lesion would have emerged after the SCI (Fig. 5). Emergence of similar axons was reported in animals after the subhemisection at C6 by the administration of antibody against MAG which is released from glial cells and prevents neural regeneration (Nakagawa et al., 2015). The present study revealed that the long-distance generation of axon branches spanning the C4/C5 segment to the C8 segment could have occurred spontaneously in the long-term by rehabilitative training.

### CST projections from ipsilesional M1

On the other hand, there was also a considerable increase in axons and bouton-like swellings originating from the ipsilesional M1, in the ipsilesional gray matter of the C8 segments spanning Lamina IX (Figs. 7*C*, 8*B*, ②). But the boutons decreased in the ipsilesional Lamina VIII and contralesional Lamina VII (Fig. 8*B*, ③). The normal CST projections are directed to the contralateral Laminae VI/VII and IX and ipsilateral Lamina VIII after recrossing (Kuypers, 1982; Armand et al., 1997). Previous studies revealed spinal commissural neurons in the Lamina VIII, which cross the midline to the contralateral side, terminate on interneurons in Lamina VII or motoneurons in Lamina IX, and play critical roles in left-right coordination (Silos‐Santiago and Snider, 1992; Eide et al., 1999; Stokke et al., 2002; Matsuyama et al., 2006; Abbinanti et al., 2012). Therefore, the decrease in the number of terminals in Lamina VII on the contralesional side and in Lamina VIII on the ipsilesional side (Fig. 8*B*, ③), and the increase in the number of axons in Laminae V and IX and that in number of terminals in Laminae VI and IX on the ipsilesional C8 (Fig. 8*B*, ②) suggest that the descending control by the ipsilesional M1 might have partly switched from control of the contralesional hand to that of the ipsilesional hand. Behaviorally, we did not observe any decline in the dexterity of the contralesional (intact side) hand movements, presumably because of redundancy in the intact motor system. The mechanism of such switch is unclear, but should be an interesting target of future studies. All these observations were already observed in the monkey Di at five months after the SCI and summarized in Figure 8*B*.

### Comparison with hemisection/subhemisection model

It is important to compare the results in the monkeys with C4/C5 DLF lesion that showed prominent recovery (Sasaki et al., 2004; Nishimura et al., 2007, 2009; Sugiyama et al., 2013; Sawada et al., 2015; Chao et al., 2019) to the monkeys with larger lesions with poorer recovery (Galea and Darian-Smith, 1997; Rosenzweig et al., 2010). In the hemisection model, extension of the recrossing CST axons from those descending in the contralesional side was found, but changes in the other components were not described. On the other hand, in the subhemisection model by Nakagawa et al. (2015) treated with anti-MAG antibody, the CST axons were extended, descended in the spinal gray matter bypassing the lesion and were connected to the motor nuclei (like Fig. 8*B*, ①). These animals showed prominent recovery in grasping. Thus, reconnection of the CST axons to the motoneurons might be one of the critical components for prominent recovery of hand dexterity.

After the full recovery of precision grip, the contralesional M1 retained the axons and terminals rostral to the lesion, presumably involving the control of C3–C4 PNs, and increased the direct control of caudal segments by extending axons that descend in the gray matter. In addition, the ipsilesional M1 increased recrossing axons caudal to the lesion, suggesting shift of its control from the intact hand to the affected hand. Thus, although previous studies suggest that the contribution of C3–C4 PNs (Tohyama et al., 2017) and the ipsilesional M1 (Nishimura et al., 2007) may decrease in the late stage of recovery, this does not always indicate that their role was completely replaced. Instead, CSTs originating from both the contralesional and ipsilesional M1 still contribute to recovery after the SCI through monosynaptic and/or multisynaptic pathways to motoneurons (Ninomiya et al., 2022), as suggested by massive reorganization of cortical networks (Nishimura et al., 2007; Nardone et al., 2013; Suzuki et al., 2020). Altogether, the results of the present study showed that interplay of the direct cortico-motoneuronal pathways originating from bilateral M1 and indirect pathway through the PNs, which were mostly transected in the hemisection model, would be the key for recovery of dexterous finger movements. Thus, the brain distributes its control over a variety of neural systems for long-term recovery. This strategy may assure robustness of motor control in the brain with a partial lesion, to avoid fatal damage induced by additional injury. Furthermore, in this study, we focused on the change in the axonal trajectories and terminations of the CST. However, it is highly likely that other descending motor systems such as rubrospinal and reticulospinal tracts contribute to long-term recovery, which should be the subject of future studies.

And in the end, we have to point out that the DLF in nonhuman primates contains the CST axons not only from the M1 but from other motor-related areas such as supplementary, premotor and cingulate motor cortex, somatosensory and parietal cortices (Toyoshima and Sakai, 1982; Galea and Darian-Smith, 1994). The functions of the CST originating from thesenon-M1 areas are mostly unclear yet. Therefore, how the CST originating from these areas would affect the recovery process would be an issue to be tackled in the future.

## Synthesis

Reviewing Editor: Benjamin Deneen, Baylor College of Medicine Decisions are customarily a result of the Reviewing Editor and the peer reviewers coming together and discussing their recommendations until a consensus is reached. When revisions are invited, a fact-based synthesis statement explaining their decision and outlining what is needed to prepare a revision will be listed below. The following reviewer(s) agreed to reveal their identity: NONE. Note: If this manuscript was transferred from JNeurosci and a decision was made to accept the manuscript without peer review, a brief statement to this effect will instead be what is listed below.

This is a transferred manuscript that was previously reviewed and the response to reviewers comments is sufficient to warrant publication in eNeuro. The first reviewer was generally quite positive and supportive, while the second reviewer raised substantive issues of data interpretation and subjective issues of novelty that were sufficiently addressed in the revised manuscript. Overall, this manuscript will make a nice contribution to eNeuro.
